# Using Sacrificial Cell Spheroids for the Bioprinting of Perfusable 3D Tissue and Organ Constructs: A Computational Study

**DOI:** 10.1155/2019/7853586

**Published:** 2019-05-20

**Authors:** Andreea Robu, Vladimir Mironov, Adrian Neagu

**Affiliations:** ^1^Department of Automation and Applied Informatics, Politehnica University of Timisoara, Timisoara 300006, Romania; ^2^3D Bioprinting Solutions, Moscow 115409, Russia; ^3^Department of Functional Sciences, Victor Babes University of Medicine and Pharmacy, Timisoara 300041, Romania; ^4^Department of Physics & Astronomy, University of Missouri, Columbia 65211, MO, USA

## Abstract

A long-standing problem in tissue engineering is the biofabrication of perfusable tissue constructs that can be readily connected to the patient's vasculature. It was partially solved by three-dimensional (3D) printing of sacrificial material (e.g., hydrogel) strands: upon incorporation in another cell-laden hydrogel, the strands were removed, leaving behind perfusable channels. Their complexity, however, did not match that of the native vasculature. Here, we propose to use multicellular spheroids as a sacrificial material and investigate their potential benefits in the context of 3D bioprinting of cell aggregates and/or cell-laden hydrogels. Our study is based on computer simulations of postprinting cellular rearrangements. The computational model of the biological system is built on a cubic lattice, whereas its evolution is simulated using the Metropolis Monte Carlo algorithm. The simulations describe structural changes in three types of tissue constructs: a tube made of a single cell type, a tube made of two cell types, and a cell-laden hydrogel slab that incorporates a branching tube. In all three constructs, the lumen is obtained after the elimination of the sacrificial cell population. Our study suggests that sacrificial cell spheroids (sacrospheres) enable one to print tissue constructs outfitted with a finer and more complex network of channels than the ones obtained so far. Moreover, cellular interactions might give rise to a tissue microarchitecture that lies beyond the bioprinter's resolution. Although more expensive than inert materials, sacrificial cells have the potential to bring further progress towards the biofabrication of fully vascularized tissue substitutes.

## 1. Introduction

Tissue engineering is aimed at developing tissue substitutes for replacing diseased organs, restoring them, or facilitating their regeneration [1]. Along the way to clinical applications of tissue engineering, a long-standing challenge is the fabrication of large tissue constructs with a network of perfusable channels that can rapidly integrate into the patient's vasculature. This problem attracted considerable interest during the last decade [2, 3]. An optimal vascular network is highly organized, including small arteries, arterioles, capillaries, venules, and small veins. The microvasculature ensures that cells are within 200 *μ*m from the closest capillary, a distance over which diffusion is an effective transport mechanism for nutrients and gases [2].

A remarkable progress has been made in tissue engineering by three-dimensional (3D) printing of sacrificial materials (carbohydrate-glass filaments [4] or thermoreversible hydrogel, Pluronic F-127 strands [5, 6]). Upon incorporation in a cell-laden hydrogel, by molding [4] or 3D bioprinting [5, 6], the sacrificial material was gently removed within minutes and the construct was perfused with cell culture medium. Although they were able to maintain the viability of thick tissue constructs, the 3D printed perfusion channels produced so far lack the architectural complexity of the native vasculature [2].

In the present work, we propose to use sacrificial cell spheroids (sacrospheres) for the 3D bioprinting of tissue constructs with the embedded vasculature. Cells can be turned into a sacrificial material by rendering them sensitive to physical or chemical factors. For example, cells cultured in the presence of crystalline silicon (Si) nanoparticles become vulnerable to radiofrequency radiation: electrical currents induced at the nanoparticle/water interface cause Joule heating at a rate that depends linearly on nanoparticle concentration [7]. The efficacy of radiofrequency-induced hyperthermia was assessed *in vitro*, on 3T3 cell lines, and *in vivo*, on a mouse model of Lewis lung carcinoma [7]. Importantly, Si nanoparticles do not pose a threat to the host organism: eventually, they are eliminated via the kidneys [8].

Another method for the selective elimination of cells is based on photosensitizers developed for the photodynamic therapy of cancer. These compounds are nontoxic to cells in the dark. When exposed to light of a certain wavelength, they become fluorescent and generate reactive oxygen species [9]. By coupling gold nanorods with a second-generation photosensitizer, Al(III) phthalocyanine chloride tetrasulfonic acid (AlPcS_4_), Jang et al. developed a complex for the elimination of cells via dual photodynamic and photothermal therapy [9]. Transmission electron microscopy revealed that the gold nanorod‐AlPcS_4_ complex was internalized by live cells in their endosomes and lysosomes. No cytotoxicity was observed in the dark for AlPcS_4_ and for the gold nanorod‐AlPcS_4_ complex [9]. The intracellular uptake of the complex was 4-fold greater than that of AlPcS_4_ alone. The internalization of the photosensitizer is important because the cytotoxic singlet oxygen has a radius of action of the order of 0.02 *μ*m [10]. Therefore, photosensitizer molecules located in the extracellular space make little damage to the adjacent cells. Exposing cells to near infrared (810 nm wavelength) radiation resulted in heat generation and stimulated the release of the photosensitizer from the gold nanorod surface. The released AlPcS_4_ molecules regained their fluorescence and photosensitivity; subsequent exposure to red light (670 nm wavelength) triggered their phototoxicity, as shown both *in vitro* and *in vivo* [9].

Sacrificial tissue spheroids could also be created using the principles of synthetic biology [11]. Recent research led to the development of a modular library of synthetic morphogenetic driver genes able to control (separately) mammalian cell adhesion, locomotion, proliferation, and elective cell death [12]. Such a library can induce desired morphological behaviors, including cell death on command.

In this study, we employ computer simulations to demonstrate that spheroids of vascular and sacrificial cells facilitate the bioprinting of tubular constructs, as well as of bulky tissue constructs that incorporate branched channels akin to a vascular tree. This work presents computer simulations based on a Metropolis Monte Carlo (MMC) algorithm [13], slightly modified to account for spontaneous, adhesion-driven rearrangements of cells within a multicellular structure [14]. Note, however, that time is not involved in the Metropolis algorithm [13]. Although the sequence of events observed in several experiments was reproduced by MMC simulations [15, 16], one cannot prove that the number of elapsed Monte Carlo steps (MCS) and the duration of the simulated process are proportional. MMC simulations do not mimic an accurate time evolution, but they account for the tendency of cells to establish the largest number of strong bonds with the entities within reach (cells or biomaterials) [16]. Such a behavior is in accord with Steinberg's differential adhesion hypothesis (DAH), which states that cells actively seek to minimize the multicellular system's total free energy of adhesion [17]. The morphogenetic mechanism proposed in this work, cell spheroid fusion, is well characterized both experimentally and theoretically [18, 19].

The systems simulated here closely resemble the tissue constructs obtained in the experimental work of Norotte et al. [20], which used sacrificial hydrogel (agarose) cylinders to provide temporary support for the bioprinted structures and to control the lumen diameter. By comparing our results with experimental ones, we discuss the advantages of sacrificial cells over inert sacrificial materials [5, 20]. We demonstrate that the complexity of the branched tubes that emerge via sacrificial cell spheroid fusion exceeds that of the perfusion channel systems built by other methods [5, 20]. We also argue that when sacrificial cells are used, multicellular self-assembly can create features that are an order of magnitude smaller than the resolution of a microextrusion bioprinter.

## 2. Materials and Methods

The computer simulations reported here were performed using the SIMMMC software [21], based on the Metropolis Monte Carlo (MMC) algorithm [13, 22].

The computational model of the biological system was built on a cubic lattice, with site occupancy specified by a particle-type index, *σ* (*σ*=0 for cell-sized volume elements of the cell culture medium, *σ*=1 for volume elements of a hydrogel that can be invaded and remodeled by cells, and *σ*=2,3,… for various cell types comprised by the system). The lattice representation is convenient from the computational point of view, but it incorporates the assumption that cell diameters are similar for all the simulated cell types.

In the model, a multicellular configuration has an energy of adhesion described in terms of works of adhesion, *ε*_*σσ*′_, defined as the energy needed to break up the bond between two neighbors of types *σ* and *σ*′. Here, the term “neighbor” refers to the set of 6 nearest, 12 next-nearest, and 8 second-nearest neighbors of a lattice site. A particle is considered to interact with its neighbors, and their energy of interaction depends solely on their type [21]. The system's total energy of adhesion can be expressed as a sum between an irrelevant constant term and the interfacial energy [22]:

(1) $$\begin{array}{c} E=\underset{\underset{\sigma <\sigma {}^{\prime }}{\sigma ,\sigma {}^{\prime }}}{\sum }\gamma_{\sigma \sigma {}^{\prime }}N_{\sigma \sigma {}^{\prime }}, \end{array}$$

where *γ*_*σσ*′_=0.5(*ε*_*σσ*_+*ε*_*σ*′*σ*′_) − *ε*_*σσ*′_ is the interfacial tension and *N*_*σσ*′_ is the total number of bonds between neighbors of types *σ* and *σ*′.

The system's evolution is simulated by swapping neighbors of different types and computing the corresponding change, Δ*E*, in the total energy of adhesion. If the change is negative or zero, the swap is accepted right away; otherwise, the acceptance probability is given by exp((−Δ*E*)/*E*_T_), where *E*_T_ is the biological analog of the energy of thermal fluctuations [23]. The computational algorithm employed by the SIMMMC software is described in detail in references [21, 24]. The brief description included here merely seeks to explain the significance of the model parameters given in the next section.

For visualization, we used VMD [25].

The simulations presented here required central processing unit (CPU) times of the order of several hours on desktop computers (Intel Core i5-7500 CPU at 3.40 GHz, with 16 GB random access memory and a solid state drive of 256 GB).

## 3. Results and Discussion

### 3.1. Simulations of the Formation of Tubular Tissue Constructs

**Figure 1:**
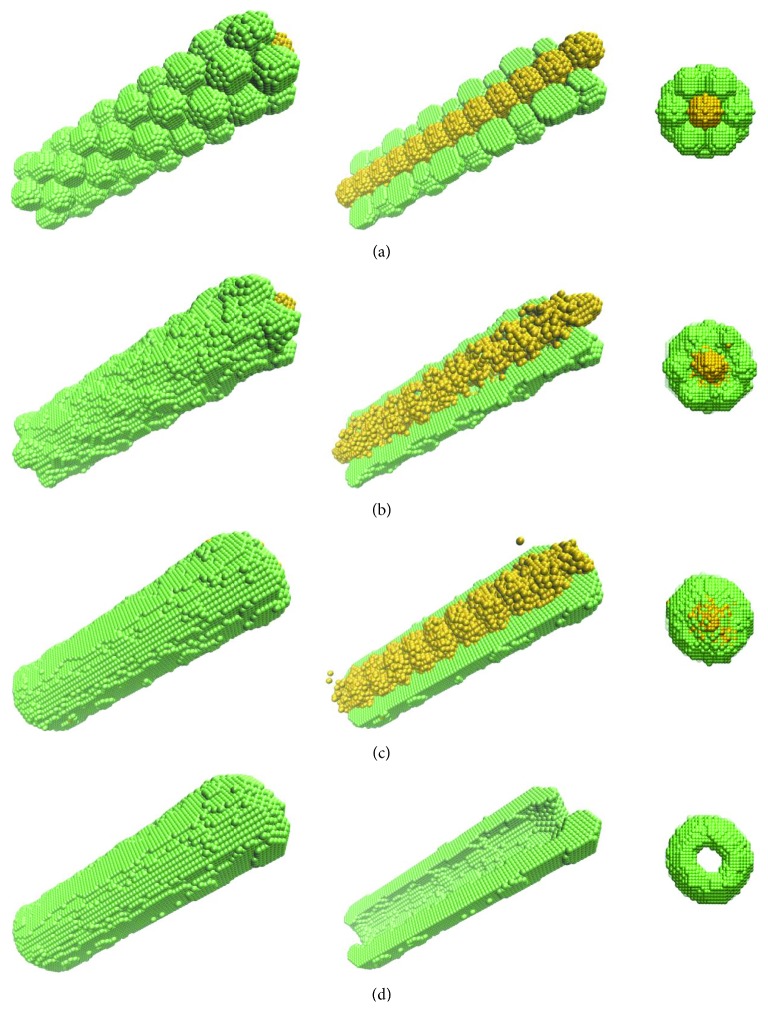
Snapshots of postprinting rearrangement of cells in a bioprinted tubular construct: the initial state (a); the state obtained within 10^3^ Monte Carlo steps (MCS) (b); the result of 5 × 10^3^ MCS, right before the elimination of sacrificial cells (yellow spheres) (c); the result of an additional 10^3^ MCS (d). The left column shows a perspective view of the whole construct, the middle column reveals the sacrificial cell population by eliminating half of the tube's wall, whereas the right column represents an axial view of the construct. Cell-cell interactions are described by the model parameters *ε*_0*σ*_=0, *σ* ∈ {0,1,2}, *ε*_11_=1.4, *ε*_12_=1.4, and *ε*_22_=1.4. Here, *σ*=0 for the cell culture medium, *σ*=1 for the desired cell type (green), and *σ*=2 for sacrificial cells (yellow).

To demonstrate the use of cells as a sacrificial material, we first consider a computational model of a bioprinted tube made of a single cell type (Figure 1). A subset of cells, represented in Figure 1 by yellow spheres, was rendered sensitive to physical factors; they can be eliminated at a desirable stage of postprinting evolution. The remaining cells, depicted as green spheres, are meant to form the wall of the tubular construct.  Figure 1(a) shows the model of the system delivered by the bioprinter: a hexagonal close-packed arrangement of cell aggregates (509 cells each, 10-cell sizes in diameter) wrapping a contiguous chain of sacrificial cell aggregates (see the central panel in Figure 1(a), in which the top half of the tube's wall is omitted to show the sacrificial cells).

Based on Steinberg's DAH [17], the computer simulation of Figure 1 shows that cells spontaneously relocate within the construct, leading to the fusion of adjacent cell spheroids. Their fusion was incomplete after 10^3^ MCS, resulting in a rough external surface of the tubular structure; this roughness disappeared after 5 × 10^3^ MCS except for the interface between normal and sacrificial cells. The rearrangement of this interface is prevented by the algorithm because the two cell populations have identical adhesivities, coming from the same cell type. Once the sacrificial cells were eliminated (after the completion of 5 × 10^3^ MCS), the internal surface of the tube became smooth within an extra 10^3^ MCS (Figure 1(d)). The rounding of the tube's extremities and the shrinking of the lumen are a result of the liquid-like behavior of the multicellular system [17], a phenomenon noticed in experiments too. The collapse of the lumen was halted as soon as the tube was connected to a perfusion bioreactor [20].

**Figure 2:**
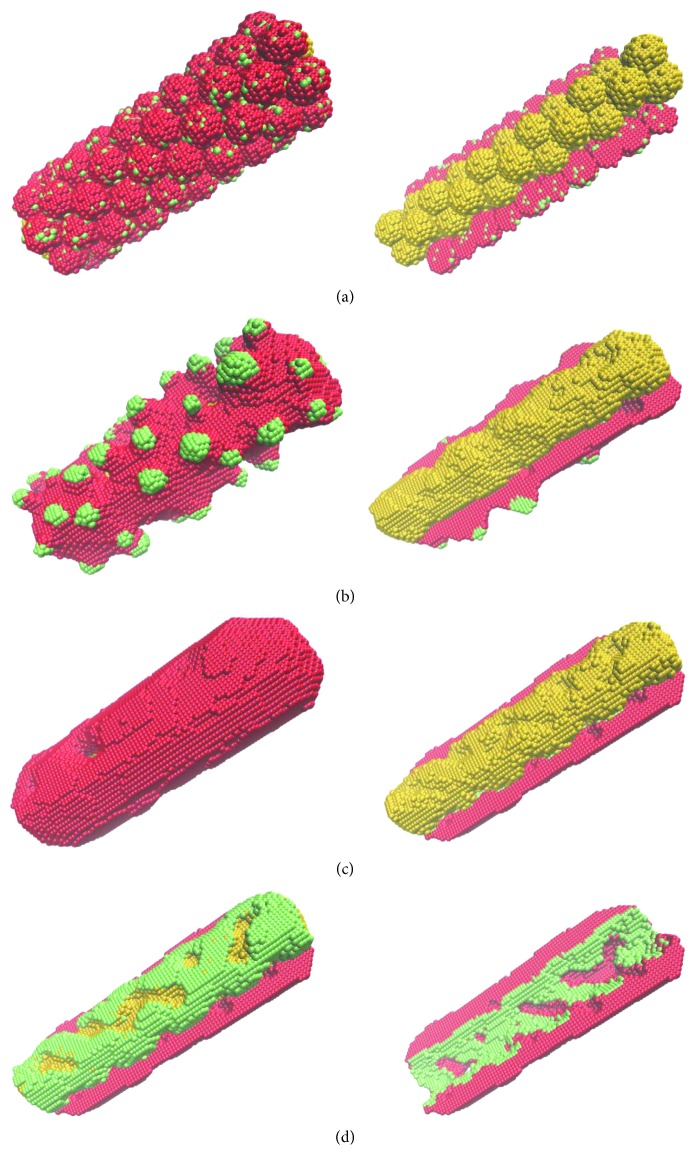
MMC simulation of the fusion of heterotypic aggregates made of randomly intermixed smooth muscle cells (red) and endothelial cells (green) and aggregates of sacrificial cells (yellow). Within 5 × 10^4^ MCS, the initial state (a) evolves into an undesired configuration (b) if the model parameters were inappropriate (*ε*_0*σ*_=0, *σ* ∈ {0,1,2,3}, *ε*_11_=1.6, *ε*_12_=1.0, *ε*_13_=1.0, *ε*_22_=2.0, *ε*_23_=1.8, and *ε*_33_=2.4) and an anatomically correct structure (c, d) if the model parameters described the right hierarchy of interfacial tensions (*ε*_0*σ*_=0, *σ* ∈ {0,1,2,3}, *ε*_11_=1.6, *ε*_12_=1.4, *ε*_13_=1.0, *ε*_22_=2.0, *ε*_23_=1.8, and *ε*_33_=2.4). Here, *σ*=0 for the cell culture medium, *σ*=1 for smooth muscle cells (red), *σ*=2 for endothelial cells (green), and *σ*=3 for sacrificial cells (yellow). In (a)–(c), the left column depicts the 3D view of the entire construct, whereas the right column exposes sacrificial cells by not showing the top half of the tube's wall. In (d), the left image shows endothelial and sacrificial cells by hiding smooth muscle cells from the top half of the tube's wall, whereas the right image shows the bottom half of the tube after the elimination of the sacrificial cells.

Figure 2 shows the simulated evolution of a bioprinted construct made of two cell types, along with a third sacrificial one.

Here, red spheres represent smooth muscle cells, green spheres stand for endothelial cells, whereas yellow spheres depict sacrificial cells. The cell aggregates shown in Figure 2 are similar in size to those in Figure 1. The ones that form the tube's wall consist of 10% endothelial cells and 90% smooth muscle cells, in a random arrangement. Such aggregates have been produced recently for 3D printing applications [26].

We assume that the 3 cell types have different adhesive properties, specified by the model parameters given in the caption of Figure 2. Their importance is illustrated in Figure 2: starting from the initial state of Figure 2(a), a simulation of 5 × 10^4^ MCS gave the configuration depicted in Figure 2(b) for one set of model parameters and that shown in Figures 2(c) and 2(d) for another set (listed in the caption of Figure 2). The latter set describes a hierarchy of interfacial tensions that cause the cell spheroids to self-assemble into a structure that resembles a blood vessel with endothelial lining. An adhesion-driven multicellular self-assembly gave rise to an incomplete endothelial layer because the cell culture medium trapped between the tube wall and the sacrificial cell population prevented their interaction. Such a behavior, however, might be a computational artifact, stemming from the inability of the MMC algorithm to describe the bulk flow of the cell culture medium. In the experimental counterpart of the system shown in Figure 2, endothelial cell proliferation and rearrangement after the start of perfusion might seal the defects. If further repair is needed, endothelial cell seeding can be employed as usual in experiments based on sacrificial hydrogel printing [5, 6].

Figure 2 demonstrates an important advantage of using sacrificial cells: the postprinting rearrangement of all cell types, governed by their adhesive properties, has the potential of creating a tissue microarchitecture of single-cell resolution. Although the resolution of an extrusion-based bioprinter is of the order of 0.1 mm [27], the directed self-assembly of the multicellular structure delivered by the printer can give rise to features that are one order of magnitude smaller.

### 3.2. Branching Tubes

Cell aggregates can be incorporated in hydrogels contiguously, enabling their subsequent fusion [15, 26]. Recently developed bioassembly instruments are able to deliver spherical microtissues, hydrogels, and scaffold materials in a precise, layer-by-layer approach, building thick tissue constructs [28].

**Figure 3:**
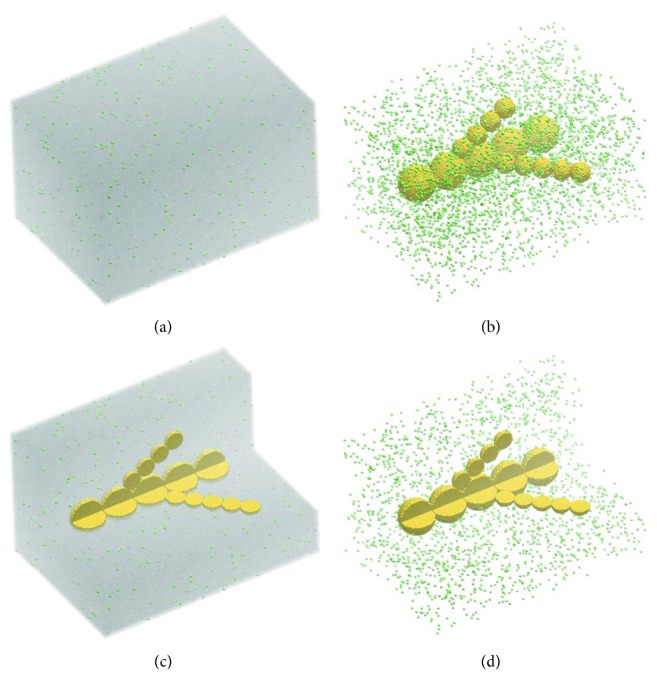
The initial state of computer simulations of a tissue construct made of endothelial (green) cells randomly dispersed in a hydrogel (10^6^ cells/mL) and a branched chain of aggregates of sacrificial (yellow) cells. The 3D view of the construct is shown in the presence (a) and in the absence (b) of the embedding hydrogel; also, part of the construct is omitted to reveal the axial cross section of the sacrificial aggregates in the presence (c) and absence (d) of the hydrogel.

**Figure 4:**
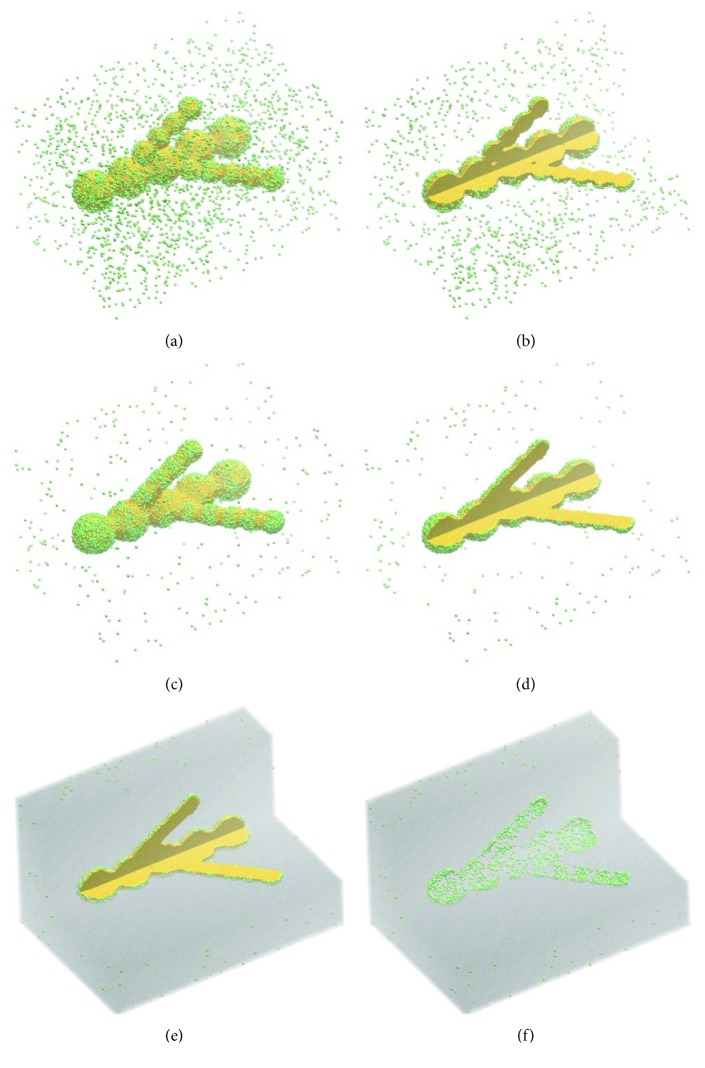
The simulated evolution of the model system in Figure 3 when the interaction energies are described by the following set of works of adhesion: *ε*_0*σ*_=0, *σ* ∈ {0,1,2,3}, *ε*_11_=2.4, *ε*_12_=2.0, *ε*_13_=2.0, *ε*_22_=1.6, *ε*_23_=2.0, and *ε*_33_=4.0; here, *σ*=0 for the cell culture medium, *σ*=1 for the hydrogel that serves as the dispersing medium for endothelial cells, *σ*=2 for endothelial cells (green), and *σ*=3 for sacrificial cells (yellow). Shown are snapshots of the emergent configurations obtained by running 5 × 10^3^ MCS (a, b) and 10^5^ MCS (c–f).

In this section, we explore the potential of such bioassembly techniques employed in conjunction with sacrificial cell spheroids.  Figure 3 shows the initial state of the simulations, made of a cell-laden hydrogel that incorporates a branched arrangement of sacrificial cell spheroids of different sizes. The large aggregates are of 30-cell sizes, and the small ones are of 16-cell sizes in diameter; such aggregate sizes are typical in bioprinting [20, 26, 29]. They are embedded in a hydrogel that contains 10^6^ endothelial cells per mL, as usual in biofabrication [5]. The system presented in Figure 3 consists of about 3.78 million particles. Assuming a cell diameter of the order of 10 *μ*m, it corresponds to an experimental system of 2.1 mm × 1.45 mm × 1.24 mm in size. Volume elements of the hydrogel are represented by silver points in Figures 3(a) and 3(c) and in Figures 4(e) and 4(f). Endothelial cells are depicted as green spheres in Figures 3 and 4. To reveal the cells, the hydrogel is hidden in Figures 3(b) and 3(d) and in Figures 4(a)–4(d).

Shown in Figure 4 is the postprinting evolution of the system depicted in Figure 3. This simulation, of 10^5^ MCS, describing the evolution of a system of realistic size, had a CPU time of about 17 hours on a desktop computer (described in Materials and Methods).

Figure 4 demonstrates that the bioprinted construct evolves into a branched structure with a contiguous lumen generated as the sacrificial cells are eliminated. The neighboring aggregates of sacrificial cells fuse within a few thousand MCS. Nevertheless, 5 × 10^3^ MCS were insufficient for a firm connection of the side branches (Figure 4(b)). As fusion evolves, within 10^5^ MCS, the side branches become fully connected to the main branch (Figure 4(d)). Endothelial cell motility and the appropriate adhesive properties lead to the accumulation of endothelial cells on the interface between the hydrogel and the sacrificial cell population (Figures 4(c)–4(e)). After sacrificial cell elimination, a branched tube results with endothelial cell lining, as shown in the cross-sectional image of Figure 4(f). If the resulting endothelial layer is not contiguous, such as in Figure 4(f), the defects might be sealed via cell proliferation or cell seeding (by perfusing the lumen with a suspension of endothelial cells [2, 5, 6]).

Multicellular spheroids are currently used in tissue engineering, being the subject of experimental [15, 30–32] and theoretical [18, 33–35] investigations. Hence, the idea of using cell spheroids to build tubular structures is not new. Spheroids and cylinders made of living cells were used as building blocks of vascular tissue constructs in a scaffold-free bioprinting approach [20]. The fusion of building blocks gave rise to smooth tubes that wrapped agarose cylinders. The latter was used as a sacrificial material meant to prevent the collapse of the lumens. Once the construct became sturdy enough, the agarose cylinders were removed and the construct was transferred into a pulsatile-flow bioreactor for maturation [20].

The use of sacrificial cell aggregates for the biofabrication of tissue constructs with the incorporated lumen has several advantages over the use of sacrificial hydrogels. Perhaps, the most important of them is the gain in resolution, ensured by the ability of living cells to relocate via mechanisms known from developmental biology. Using cell aggregates of 100–170 *μ*m in diameter [26], their subsequent fusion can lead to lumen diameters in the range of 90–150 *μ*m. By contrast, printing a continuous thread of Pluronic F-127, upon removal by cooling to 4°C, gave rise to hollow channels of about 500 *μ*m in diameter [5]. Indeed, the printability of hydrogels depends on a delicate balance of their rheological parameters, which are thermally tunable, but within limits imposed by the need to preserve cell viability [36]. Using drop-on-demand printers, one is able to deliver individual cell aggregates in precise locations, thereby improving the resolution 3-fold. If, additionally, cells rearrange according to DAH [17], the emergent structure might have features of the order of 10 *μ*m in size, such as the monolayer of endothelial cells observed in Figures 2 and 4.

Another advantage of using sacrificial cell aggregates is the ability to print tissue constructs that incorporate a complex architecture of branched tubes of 0.1 to 1 mm in diameter. Moreover, sacrificial cell spheroids could be combined with prevascularized spheroids, developed recently [26]. In spheroids composed of endothelial cells, fibroblasts, and adipose tissue-derived mesenchymal stem cells, the endothelial cells gave rise to structures similar to capillary vessels. Aggregates placed contiguously fused after one day, and a capillary-like network emerged throughout the construct within 4 days [26]. Thus, in combination with sacrificial cell aggregates, prevascularized spheroids might enable the biofabrication of a complete vascular tree with diameters of branched vascular segments ranging from those of small arteries to capillaries.

Finally, spheroids of cells sensitized to physical or chemical factors represent the handiest sacrificial material to be used with bioprinters designed to deliver cell aggregates [37]. Among them, the Regenova robot (Cyfuse, Japan), which inserts cell aggregates into a set of stainless steel microneedles, could only use cell aggregates as a sacrificial material (the needles obstruct the access of a print head needed to deliver a thread of inert sacrificial material) [29]. Actually, the Regenova platform does not require any sacrificial material for building multicellular tubes as small as 0.5 mm in diameter [38]. Sacrificial spheroids might extend the capabilities of cell aggregate bioprinters, enabling the biofabrication of branching channels with lumens of the order of 0.1 mm in diameter.

It is interesting that cell death (apoptosis) is a natural mechanism of vascular development [39]. Thus, our approach using sacrificial cells could be considered biomimetic.

Nevertheless, the methodology proposed in this work is not free from drawbacks. Using sacrificial cell spheroids is expensive: high costs are involved in finding a suitable cell source, expanding the cells, making them sensitive to the factors that will be used to eliminate them, and producing a large number of spheroids similar in size and shape, suitable for 3D printing. Furthermore, our method relies on computational modeling for predicting the postprinting evolution. While modeling is useful in most forms of bioprinting [22], here it is vital to ensure that, in the early stages of tissue fusion, no portion of the construct will suffer from lack of oxygen and nutrients. The design of the initial construct should take into account postprinting fusion to ensure that all the cells of interest are at most 0.2 mm away from the cell culture medium [2]. Sacrificial cells might be buried deeper into the construct, but in the absence of proper gas and nutrient exchange, their motility will decrease, slowing down the fusion of adjacent spheroids. Hence, computational modeling is essential if one plans to use cells as a sacrificial material.

## 4. Conclusions

The present study proposes to use sacrificial cells for the biofabrication of perfusable tissue constructs. Our approach relies on tissue fusion, a robust morphogenetic mechanism known from developmental biology. The potential of the proposed 3D printing methodology is investigated via computer simulations based on the Metropolis Monte Carlo algorithm, known to describe adhesion-driven rearrangements of cells within engineered tissues [16, 22].

Our study demonstrates the potential of using multicellular spheroids as a sacrificial material in the 3D bioprinting of vascularized tissue constructs. The simulations pointed out the advantages of sacrificial cells compared to sacrificial materials used so far: (i) cell spheroids fuse into threads that are finer and more complex than inert material threads obtained by extrusion bioprinting and (ii) spontaneous rearrangements of cells in heterotypic multicellular structures can lead to feature sizes of the order of one cell diameter, beyond the resolution of most bioprinters. Our simulations also revealed hierarchies of cell-cell interaction energies needed for obtaining small diameter tubes made of one or two cell types, as well as branched tubes within a bulky construct made of cell-laden hydrogel.

Taken together, our simulations suggest that sacrificial cell spheroids and prevascularized multicellular spheroids might be employed for bioprinting tissue constructs outfitted with perfusable channels ranging from macrovasculature to arterioles and venules.

## Data Availability

The computer simulation data that form the basis of this study are available from the corresponding author upon request.
